# Machine learning-based investigation of the relationship between immune status and left ventricular hypertrophy in patients with end-stage kidney disease

**DOI:** 10.3389/fcvm.2023.1187965

**Published:** 2023-05-18

**Authors:** Min Yang, Bo Peng, Quan Zhuang, Junhui Li, Pengpeng Zhang, Hong Liu, Yi Zhu, Yingzi Ming

**Affiliations:** ^1^ Transplantation Center, The Third Xiangya Hospital, Central South University, Changsha, China; ^2^ Engineering and Technology Research Center for Transplantation Medicine of National Health Commission, Changsha, China

**Keywords:** immune state, ESKD, LVH, machine learning, immune function monitoring

## Abstract

**Background:**

Left ventricular hypertrophy (LVH) is the most frequent cardiac complication among end-stage kidney disease (ESKD) patients, which has been identified as predictive of adverse outcomes. Emerging evidence has suggested that immune system is implicated in the development of cardiac hypertrophy in multiple diseases. We applied machine learning models to exploring the relation between immune status and LVH in ESKD patients.

**Methods:**

A cohort of 506 eligible patients undergoing immune status assessment and standard echocardiography simultaneously in our center were retrospectively analyzed. The association between immune parameters and the occurrence of LVH were evaluated through univariate and multivariate logistic analysis. To develop a predictive model, we utilized four distinct modeling approaches: support vector machine (SVM), logistic regression (LR), multi-layer perceptron (MLP), and random forest (RF).

**Results:**

In comparison to the non-LVH group, ESKD patients with LVH exhibited significantly impaired immune function, as indicated by lower cell counts of CD3^+^ T cells, CD4^+^ T cells, CD8^+^ T cells, and B cells. Additionally, multivariable Cox regression analysis revealed that a decrease in CD3^+^ T cell count was an independent risk factor for LVH, while a decrease in NK cell count was associated with the severity of LVH. The RF model demonstrated superior performance, with an average area under the curve (AUC) of 0.942.

**Conclusion:**

Our findings indicate a strong association between immune parameters and LVH in ESKD patients. Moreover, the RF model exhibits excellent predictive ability in identifying ESKD patients at risk of developing LVH. Based on these results, immunomodulation may represent a promising approach for preventing and treating this disease.

## Background

End-stage kidney disease (ESKD) is a growing global health concern (1). Left ventricular hypertrophy (LVH) is the most common cardiac abnormality and an adverse prognostic indicator for clinical cardiovascular outcomes including morbidity and mortality in ESKD patients (2–5). Although numerous studies have revealed that hypertension, fluid overload, oxidative stress and chronic inflammation directly or indirectly impinge upon cardiac hypertrophy and remodeling in ESKD patients (4, 6, 7), the pathogenesis of LVH is complicated and not fully understood yet. Emerging evidence suggests that involvement of immune system is associated with the development and progression of cardiac hypertrophy and remodeling (8–10). Furthermore, impaired immune function has been described in ESKD patients, attributed to factors such as uremia and lymphocyte apoptosis (11, 12). Therefore, immune cells may play critical roles in LVH, potentially providing a novel therapeutic target for prevention and treatment.

Immune function monitoring is a simple and effective to reflect the immune status, including the proportion and cell counts of circulating immune cell subsets. Recent studies have demonstrated that circulating immune cell subsets (T, B, NK cells) are associated with cardiac hypertrophy and remodeling in various pathological conditions. For instance, in pressure overload-induced cardiac hypertrophy, CD4^+^ T cells including Th1 and Th17 cells play dominant roles in the process with different subsets exerting diverse influences (9, 10). In animal models of uremic cardiomyopathy, systemic accumulation of proinflammatory T cells has been shown to cause cardiac hypertrophy and remodeling (13). In ischemic cardiomyopathy animal model, regulatory T-lymphocytes dysfunction is tightly linked to adverse cardiac remodeling (14, 15). B lymphocytes have been found to be associated with cardiac hypertrophy and remodeling in ESKD patients (16). Additionally, the activation of NK cell plays critical roles in cardiac remodeling after myocardial infarction (17). Nevertheless, analyzing single immunological indicator may not be sufficient to capture the full picture of immune status.

Machine Learning (ML) is a powerful tool that can identify patterns in large sets of data to generate predictive models with greater precision. Traditional statistical methods may be limited in their ability to capture the complexities of large datasets, particularly those with high dimensionality or nonlinear relationships. In our study, we have chosen to employ a machine-learning-based approach due to the inherent complexity of the immune system and the need to analyze a large number of variables simultaneously (18). The identification of biomarkers associated with immune function requires the integration of multiple variables and the exploration of complex relationships between them, which are ideally suited to machine learning methods. Our previous investigations have demonstrated that applying ML models is conducive to improving prognosis of renal transplantation recipients with infective complications and acute-on-chronic liver failure patients following liver transplantation (19, 20). Therefore, the application of ML may help us better understand the association of immune cells and cardiac hypertrophy and remodeling in ESKD.

In this study, we retrospectively analyzed the immune monitoring results of ESKD patients in our center and applied ML models to explore the association between these results and LVH in ESKD patients.

## Methods

### Study design and population

The retrospective, case–control study recruited 825 ESKD patients were recruited from the Transplantation Center, The Third Xiangya Hospital, Central South University between July 1, 2019, and December 31, 2021. The patients who underwent immune status assessment and standard echocardiography simultaneously were included in the study. The exclusion criteria included: (1) history of solid organ transplantation; (2) history of infection within a week; (3) history of malignant tumor. The study was reviewed and approved by the Institutional Review Board of Third Xiangya Hospital, Central South University (No. 22255).

### Definition of LVH and its severity

Experienced physicians assessed left ventricular internal diameter (LVID), ventricular septal thickness (VST), posterior wall thickness (PWT) and left ventricular end diastolic diameter (LVEDD) from echocardiography M-mode tracings. From these measurements, left ventricular mass (LVM) was calculated using the formula 0.8 × 1.04 × [(LVID + VST + PWT)3–LVEDD3] + 0.6. The LVM index (LVMI) was then determined as the ratio of LVM to body surface area. The presence of left ventricular hypertrophy (LVH) was determined based on an LVMI greater than 115 g/m^2^ in men and greater than 95 g/m^2^ in women. Based on the LVMI, the severity of LVH was classified as mild, moderate, or severe. Mild LVH was defined as an LVMI between 96 and 108 g/m^2^ for women and between 116 and 131 g/m^2^ for men. Moderate LVH was defined as an LVMI between 109 and 121 g/m^2^ for women and between 132 and 148 g/m^2^ for men. Severe LVH was defined as an LVMI greater than 121 g/m^2^ for women and greater than 148 g/m^2^ for men (21).

### Immune monitoring panel

The assessment of immune status was carried out using BD Multitest 6-color TBNK reagent with BD Trucount tubes, in order to determine the percentages and absolute counts of various circulating immune cell subsets, namely CD4^+^ T cells, CD8^+^ T cells, CD19^+^ B cells and NK cells. The assay protocol was carried out in accordance with the manufacturer's instructions (BD Biosciences, USA). Anticoagulant whole blood (50 µl) was introduced into BD TruCOUNT Tubes (BD Biosciences), followed by the addition of a monoclonal antibody mixture (20 µl). After incubation in the dark for 15 min, the cells were analyzed using the BD FACSCanto clinical software (BD Biosciences, San Jose, CA).

### Model building

To investigate the association between the immune status and LVH in ESKD patients, we employed four ML classifiers, namely, SVM, LR, MLP, and RF. The ML models were trained using fivefold cross-validation with Python programming language (version 3.6) and Scikit-learn package (version 0.22) (19). To evaluate their performance, k-fold cross-validation (with *k* = 5) was applied to 532 patients. The patients were randomly divided into five subgroups, with one subgroup used for validation and the remaining four for derivation. The appropriate hyperparameters were selected, and the average area under the curve (AUC) was computed through five independent runs. The final algorithms were developed using the full dataset of the eligible group. The RF model, which included ten trees, was trained on a diverse portion of the database and predictions were obtained through majority voting.

### Statistical analysis

Continuous data were presented as mean ± standard deviation (SD) and compared using appropriate tests such as Student's *t*-test, Welch's *t*-test, or the Mann–Whitney *U* test. Categorical data were compared using Pearson's chi-squared (*χ*^2^) test or Fisher's exact test, as appropriate. The associations between immune status and LVH and its severity were analyzed using univariate, multivariate, and multiple ordinal logistic regression analysis. The performance of the models was evaluated by calculating the AUC of the receiver operating characteristic (ROC) curve and The ROC curves were compared using the DeLong test. The statistical analysis was conducted using SPSS version 22.0 (SPSS, Inc., Chicago, IL, USA) and GraphPad Prism 9.0, and a *P*-value of <0.05 was considered statistically significant.

## Results

### Patient characteristics

**Figure 1:**
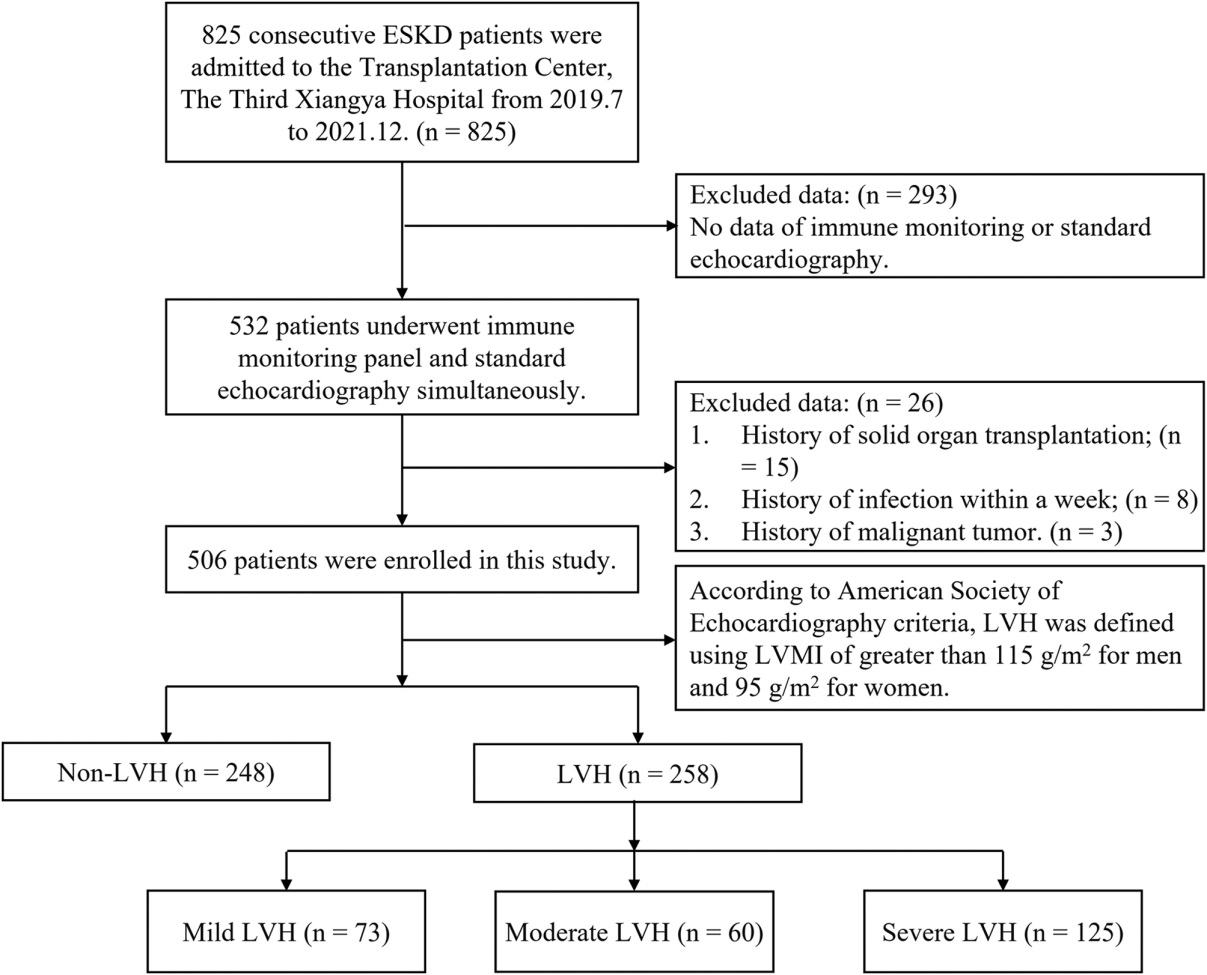
The study flow and exclusion criteria. 506 ESKD patients with immune monitoring panel and standard echocardiography simultaneously were enrolled. Based on the definition for LVH, patients were classified into either the LVH group or non-LVH group. The LVH group was further subdivided into three subgroups based on the severity of LVH: mild (*n* = 74), moderate (*n* = 60), and severe (*n* = 125). ESKD, end-stage kidney disease; LVMI, Left ventricular mass index; LVH, left ventricular hypertrophy.

In our center, a total of 532 patients with ERSD underwent immune status assessment and standard echocardiography from July 2019 to December 2021. After excluding 26 patients, 506 patients were enrolled for data analysis. According to American Society of Echocardiography (ASE) criteria, 258 patients of them were classified into the LVH subgroup. We further subdivided the LVH subgroup into mild (*n* = 74), moderate (*n* = 60), and severe (*n* = 125) LVH subgroups based on LVMI. The study flow and criteria are depicted in Figure 1.

Table 1 summarized the clinical characteristics of the LVH and non-LVH groups. The LVH group exhibited lower hemoglobin, white blood cell counts, neutrophil counts, and lymphocyte counts than the non-LVH group (*P* = 0.001, all). Additionally, the LVH group had longer dialysis time (*P* = 0.007), as well as higher systolic (*P* = 0.002) and diastolic (*P* < 0.001) blood pressure. The use of ACEI (angiotensin converting enzyme inhibitions) or ARB (angiotensin receptor blockers) was also more prevalent in the LVH group (*P* < 0.001). As expected, the LVH group had higher LVID (5.29 ± 0.62 vs. 4.61 ± 0.52), VST (1.26 ± 0.20 vs. 1.05 ± 0.18), PWT (1.20 ± 0.22 vs. 0.98 ± 0.15), LVM (241.90 ± 62.18 vs. 147.23 ± 35.69), and LVH (145.41 ± 31.81 vs. 87.43 ± 16.69) than the non-LVH group (*P* < 0.001, all) consistent with the definition of LVH. However, there was no statistical difference in age, sex, body mass index (BMI), diabetes, hemodialysis and antihypertensive drug use between the two groups.

**Table 1:** Clinical characteristics of the LVH and Non-LVH group in ESKD patients.

Parameters	All (*n* = 507)	LVH (*n* = 258)	Non-LVH (*n* = 248)	*P-*value
Age, years ± SD	41.20 ± 11.22	41.40 ± 11.41	41.00 ± 11.04	0.70
Male, *n* (%)	362 (71.4%)	176 (68.2%)	186 (75.0%)	0.09
BMI, (Kg/m^2^)	22.98 ± 4.27	22.75 ± 3.67	23.22 ± 4.82	0.22
Diabetes, *n* (%)	50 (9.9%)	24 (9.3%)	26 (10.5%)	0.66
Hemodialysis, *n* (%)	325 (64.1%)	174 (67.4%)	151 (60.9%)	0.12
Dialysis time (months), mean ± SD	16.07 ± 25.47	19.09 ± 29.12	12.94 ± 20.62	0.007
Systolic blood pressure (mmHg), mean ± SD	144.72 ± 19.72	148.65 ± 20.18	140.64 ± 20.18	<0.001
Diastolic blood pressure (mmHg), mean ± SD	92.49 ± 13.13	94.24 ± 12.84	90.67 ± 13.19	0.002
Antihypertensive drug use, *n* (%)	483 (95.3%)	249 (96.5%)	234 (94.4%)	0.29
ACEI or ARB use, *n* (%)	386 (76.1%)	217 (84.1%)	169 (68.1%)	<0.001
LVID (cm), mean ± SD	4.96 ± 0.69	5.29 ± 0.63	4.61 ± 0.57	<0.001
VST (cm), mean ± SD	1.16 ± 0.21	1.26 ± 0.20	1.05 ± 0.18	<0.001
PWT (cm), mean ± SD	1.10 ± 0.22	1.20 ± 0.22	0.98 ± 0.15	<0.001
LVD (cm), mean ± SD	195.50 ± 69.53	241.90 ± 62.18	147.23 ± 35.69	<0.001
LVH(g/m^2^), mean ± SD	117.00 ± 38.64	145.41 ± 31.81	87.43 ± 16.69	<0.001
Hb (g/L), mean ± SD	105.80 ± 22.75	102.40 ± 23.23	109.34 ± 21.72	0.001
WBC (10^9^/L), mean ± SD	6.41 ± 2.20	6.09 ± 2.20	6.74 ± 2.15	0.001
Lym (10^9^/L), mean ± SD	1.21 ± 0.47	1.14 ± 0.42	1.28 ± 0.51	0.001

Comparison between the LVH and non-LVH group.

ESKD, end-stage kidney disease; LVH, left ventricular hypertrophy; SD, standard deviation; BMI, Body mass index; ACEI, angiotensin converting enzyme inhibitions; ARB, angiotensin receptor blockers; LVMI, Left ventricular mass index; LVID, Left ventricular internal diameter; VST, ventricular septal thickness; PWT, posterior wall thickness; LVD, left ventricular end diastolic diameter (LVEDD); LVM, Left ventricular mass; Hb, hemoglobin; WBC, white blood cell; Lym, lymphocyte.

### Immune status in LVH patients with ERSD

**Figure 2:**
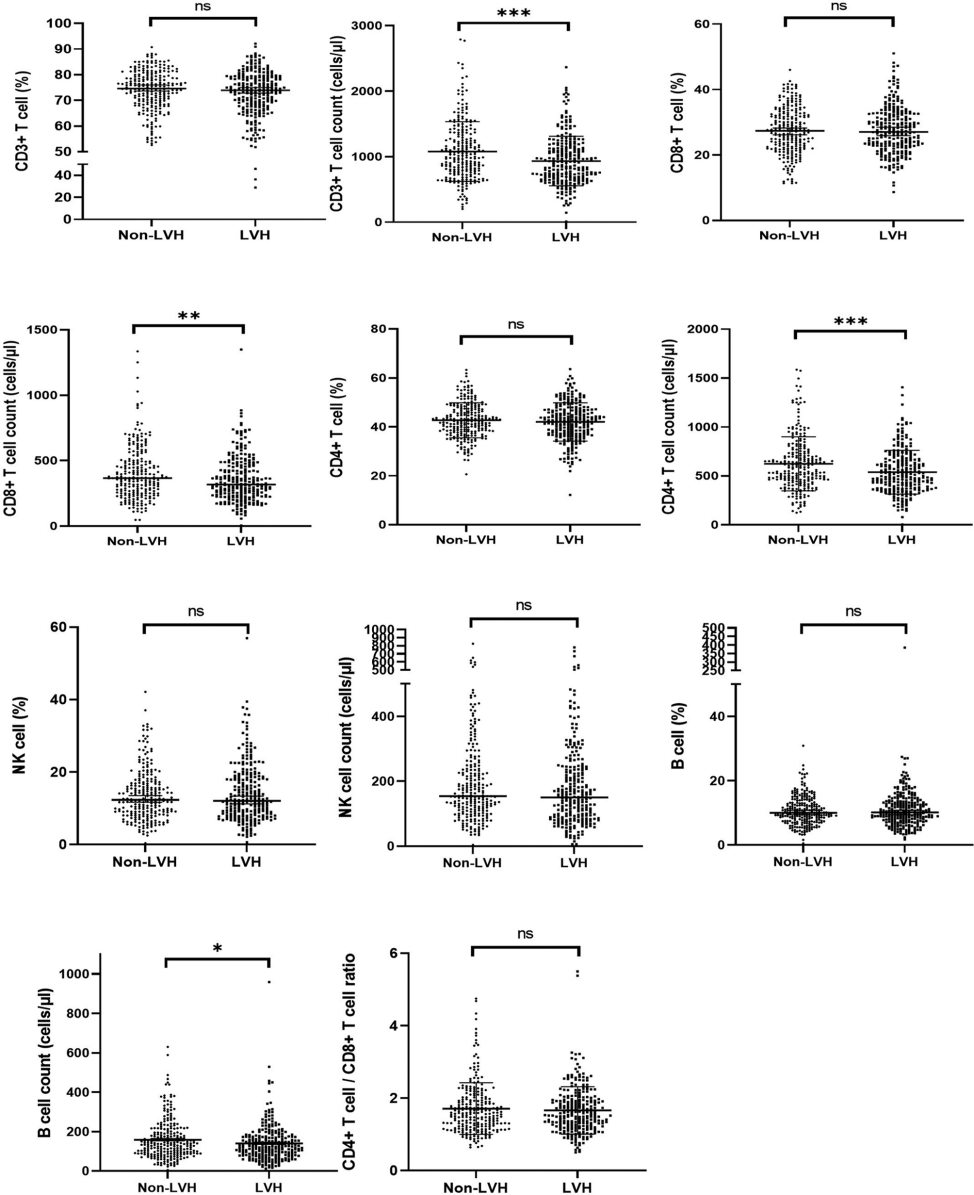
The proportion and cell counts of circulating immune cells in the LVH and Non-LVH group in ESKD patients. ESKD, end-stage kidney disease; LVH: left ventricular hypertrophy. ∗∗∗∗ means *P* < 0.001, ∗∗ means *P* < 0.05, and ∗ means *P* < 0.1.

Table 2 and Figure 2 revealed that the LVH group exhibited a poorer immune profile compared to the non-LVH group, as evidenced by significantly lower cell counts of CD3^+^ T cells (933.57 ± 376.47 vs. 1,079.20 ± 455.72), CD4^+^ T cells (537.65 ± 223.13 vs. 623.37 ± 276.50), CD8^+^ T cells (355.35 ± 175.65 vs. 402.75 ± 203.38), and B cells (140.24 ± 95.47 vs. 158.92 ± 101.44). However, the cell counts of NK cells and the percentages of each subset did not differ significantly between the two groups. It was worth noting that the CD4/CD8 ratio, a known immune biomarker, did not show a significant difference (*P* = 0.43) between the LVH and non-LVH groups.

**Table 2:** Immune monitoring panel of the LVH and Non-LVH group in ERSD patients.

Parameters	All (*n* = 507)	LVH (*n* = 259)	Non-LVH (*n* = 248)	*P-*value
CD3^+^ T cells/TBNK, mean ± SD (%)	72.34 ± 8.47	72.78 ± 8.93	73.92 ± 7.93	0.13
CD3^+^ T cells, *n* ± SD (cells/µl)	1,004.81 ± 423.03	933.57 ± 376.47	1,079.20 ± 455.72	<0.001
CD8^+^ T cells/TBNK, mean ± SD (%)	27.52 ± 7.14	27.55 ± 7.17	27.48 ± 7.12	0.92
CD8^+^ T cells, *n* ± SD (cells/µl)	378.54 ± 191.01	355.35 ± 175.65	402.75 ± 203.38	0.005
CD4^+^ T cells/TBNK, mean ± SD (%)	42.35 ± 7.50	42.03 ± 7.81	42.69 ± 7.15	0.32
CD4^+^ T cells, *n* ± SD (cells/µl)	579.58 ± 254.06	537.65 ± 223.13	623.37 ± 276.50	<0.001
NK cells/TBNK, mean ± SD (%)	13.82 ± 7.54	14.05 ± 7.97	13.57 ± 7.07	0.48
NK cells, *n* ± SD (cells/µl)	185.88 ± 126.49	178.95 ± 125.64	193.12 ± 127.23	0.21
B cells/TBNK, mean ± SD (%)	11.61 ± 17.29	12.47 ± 23.79	10.71 ± 4.46	0.25
B cells, *n* ± SD (cells/µl)	149.38 ± 98.78	140.24 ± 95.47	158.92 ± 101.44	0.033
CD4/CD8 ratio, mean ± SD	1.69 ± 0.68	1.66 ± 0.65	1.71 ± 0.71	0.43

Comparison between the LVH and non-LVH group.

ESKD, end-stage kidney disease; LVH, left ventricular hypertrophy; NK cells, natural killer cells; TBNK, T, B, and NK cells.

### Risk factors for LVH in ERSD patients

To identify the risk factors of LVH in ERSD patients, logistic analysis was performed using the results of immune parameters. As presented in Table 3, univariate logistic analysis revealed that lower cell counts of CD3^+^ T cells (unadjusted OR 0.999; 95% CI 0.990–1.000; *P* < 0.001), CD8^+^ T cells (unadjusted OR 0.996; 95% CI 0.995–0.998; *P* = 0.006), CD4^+^ T cells (unadjusted OR 0.999; 95% CI 0.998–0.999; *P* < 0.001) and B cells (unadjusted OR 0.998; 95% CI 0.996–1.000; *P* < 0.001) were significantly associated with LVH in ERSD patients. Multivariate analysis further revealed that lower cell counts of CD3^+^ T cells (unadjusted OR 0.995; 95% CI 0.991–1.000; *P* = 0.031) were independent risk factors for LVH in ERSD patients.

**Table 3:** Univariate and multivariate odds ratios for LVH diagnosis in ERSD patients.

Parameters	Univariate analysis		Multivariate analysis	
	OR (95% CI)	*P-*value	OR (95% CI)	*P-*value
CD3^+^ T cells/TBNK, mean ± SD (%)	0.984 (0.964–1.005)	0.125		
CD3^+^ T cells, *n* ± SD (cells/µl)	0.999 (0.999–1.000)	<0.001	0.995 (0.991–1.000)	0.031
CD8^+^ T cells/TBNK, mean ± SD (%)	1.002 (0.978–1.027)	0.879		
CD8^+^ T cells, *n* ± SD (cells/µl)	0.996 (0.995–0.998)	0.006	1.005 (1.000–1.009)	0.05
CD4^+^ T cells/TBNK, mean ± SD (%)	0.988 (0.965–1.011)	0.316		
CD4^+^ T cells, *n* ± SD (cells/µl)	0.999 (0.998–0.999)	<0.001	1.003 (0.999–1.007)	0.143
NK cells/TBNK, mean ± SD (%)	1.009 (0.986–1.032)	0.462		
NK cells, *n* ± SD (cells/µl)	0.999 (0.998–1.001)	0.217		
B cells/TBNK, mean ± SD (%)	1.014 (0.982–1.047)	0.388		
B cells, *n* ± SD (cells/µl)	0.998 (0.996–1.000)	<0.001	1.001 (0.998–1.003)	0.547
CD4/CD8 ratio, mean ± SD	0.899 (0.696–1.161)	0.414		

OR, odds ratio; NK cells, natural killer cells; TBNK, T, B, and NK cells; CI, confidence interval.

### Immune status related with the severity of LVH in ERSD patients

**Figure 3:**
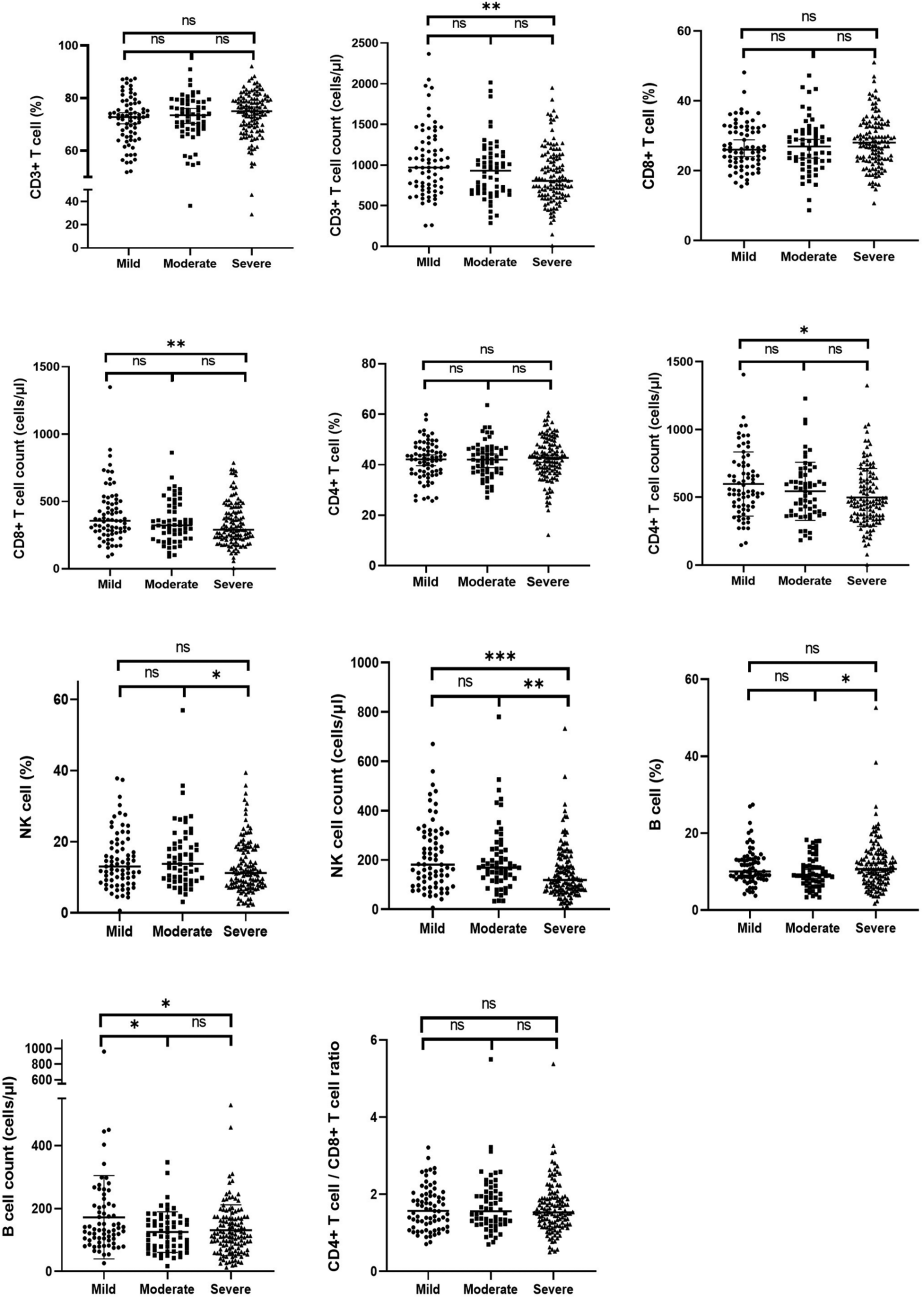
The proportion and cell counts of circulating immune cells among the mild, moderate and severe LVH group in ESKD patients. ESKD, end-stage kidney disease; LVH, left ventricular hypertrophy. ∗∗∗∗ means *P* < 0.001, ∗∗ means *P* < 0.05, and ∗ means *P* < 0.1.

The results of the immune parameters were compared across three LVH subgroups. Statistical differences were observed in the cell counts of each subset, while there were no significant differences in the percentages of each subset and the CD4/CD8 ratio (Table 4). Figure 3 presents the comparisons between each pair of subgroups, indicating that there were statistically significant differences in the cell counts of each subset between the mild and severe groups (*P* < 0.05). The moderate group had higher cell counts of NK cells but lower cell counts of B cells compared to the severe group, with both differences being statistically significant (*P* < 0.05).

**Table 4:** Immune monitoring panel in ERSD patients with different severity of LVH.

Parameters	Mild LVH (*n* = 73)	Moderate LVH (*n* = 60)	Severe LVH (*n* = 125)	*P-*value
CD3^+^ T cells/TBNK, mean ± SD (%)	71.67 ± 8.86	72.19 ± 9.04	73.66 ± 8.91	0.274
CD3^+^ T cells, *n* ± SD (cells/µl)	1,045.19 ± 419.88	933.73 ± 357.60	868.40 ± 346.51	0.006
CD8^+^ T cells/TBNK, mean ± SD (%)	27.08 ± 6.58	27.01 ± 7.62	28.15 ± 7.28	0.469
CD8^+^ T cells, *n* ± SD (cells/µl)	400.67 ± 207.40	348.72 ± 156.73	333.05 ± 160.26	0.03
CD4^+^ T cells/TBNK, mean ± SD (%)	41.47 ± 7.50	42.02 ± 7.08	42.35 ± 8.38	0.747
CD4^+^ T cells, *n* ± SD (cells/µl)	598.30 ± 236.36	544.23 ± 214.16	499.34 ± 213.67	0.01
NK cells/TBNK, mean ± SD (%)	14.74 ± 7.90	15.67 ± 8.94	12.90 ± 7.41	0.06
NK cells, *n* ± SD (cells/µl)	213.64 ± 136.19	201.18 ± 133.55	148.46 ± 108.08	0.01
B cells/TBNK, mean ± SD (%)	11.72 ± 4.74	9.78 ± 3.98	11.72 ± 6.64	0.089
B cells, *n* ± SD (cells/µl)	169.29 ± 129.50	125.08 ± 64.52	130.79 ± 80.84	0.008
CD4/CD8 ratio, mean ± SD	1.64 ± 0.56	1.73 ± 0.75	1.64 ± 0.66	0.671

Comparison among the mild, moderate and severe LVH group.

ESKD, end-stage kidney disease; LVH, left ventricular hypertrophy; NK cells, natural killer cells; TBNK, T, B, and NK cells.

**Figure 4:**
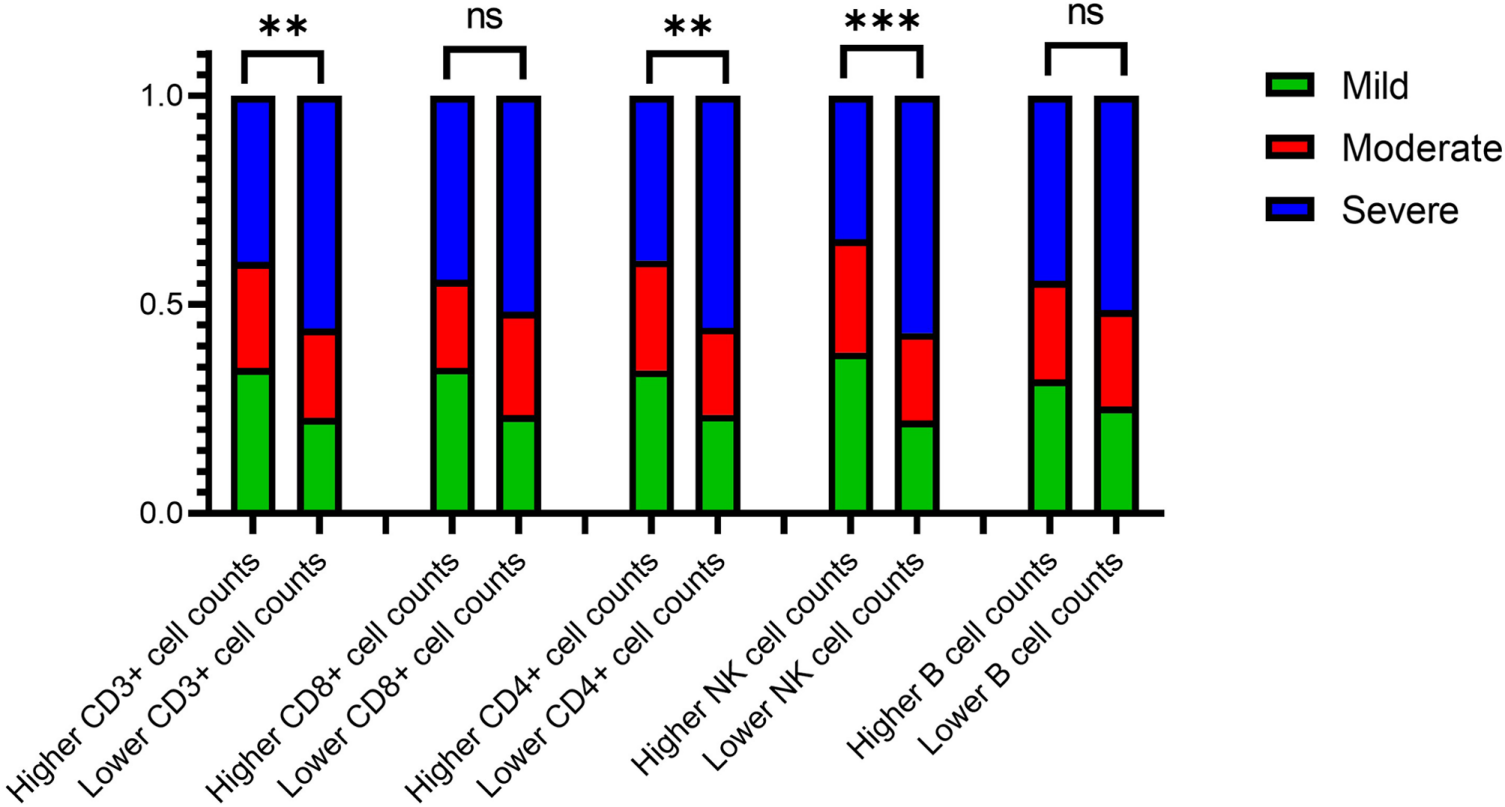
Column stacking chart of the proportion of the LVH severity in the higher or lower group of lymphocyte count. LVH, left ventricular hypertrophy. ∗∗∗∗ means *P* < 0.001, ∗∗ means *P* < 0.05, and ∗ means *P* < 0.1.

The lymphocyte subset was divided into higher and lower groups based on the median cell count values in ERSD patients with LVH. The stacked bar chart in Figure 4 shows the LVH subgroup percentages for each group. Compared to the higher groups, the lower groups had a lower frequency of mild LVH and a higher frequency of severe LVH. A significant Chi-square test demonstrated that the distribution of LVH subgroups varied in the higher and lower groups of CD3^+^ T cells, CD4^+^ T cells, and NK cells (*P* < 0.05).

The lymphocyte subset was divided into the higher and lower group by median values of cell counts in ERSD patients with LVH. The percentages of the LVH subgroup (mild, moderate and severe) were plotted in Figure 4 as stacked bar charts. Compared with the higher groups, the lower groups had respectively lower frequency of perpetration for the mild LVH and the higher for the severe LVH. A significant Chi-square test showed that the distribution of LVH subgroup frequencies varied in the higher and lower group of CD3^+^ T cells, CD4^+^ T cells and NK cells (*P* < 0.05).

To identify the risk factors of the severity of LVH, logistic regressions was performed with the cell counts of each subset, which showed statistically significant differences as presented in Table 5. The multiple ordinal logistic regressions revealed that lower cell counts of NK cells (unadjusted OR 0.997; 95% CI −0.005 – −0.001; *P* = 0.003) were independent risk factors for the severity of LVH among ERSD patients.

**Table 5:** Multiple ordinal logistic regression for the LVH severity in ERSD patients.

Parameters	B	S.E.	Wald	*P-*value	OR	95% CI
CD3^+^ T cells, *n* ± SD (cells/µl)	−0.003	0.003	0.964	0.326	0.997	−0.01–0.003
CD8^+^ T cells, *n* ± SD (cells/µl)	0.003	0.004	0.756	0.384	1.003	−0.004–0.01
CD4^+^ T cells, *n* ± SD (cells/µl)	0.003	0.003	0.61	0.435	1.003	−0.004–0.01
NK cells, *n* ± SD (cells/µl)	−0.003	0.001	8.732	0.003	0.997	−0.005–−0.001
B cells, *n* ± SD (cells/µl)	−0.001	0.002	0.729	0.393	0.999	−0.005–−0.001

OR, odds ratio; NK cells, natural killer cells; CI, confidence interval.

### Machine learning models based on immune monitoring

**Figure 5:**
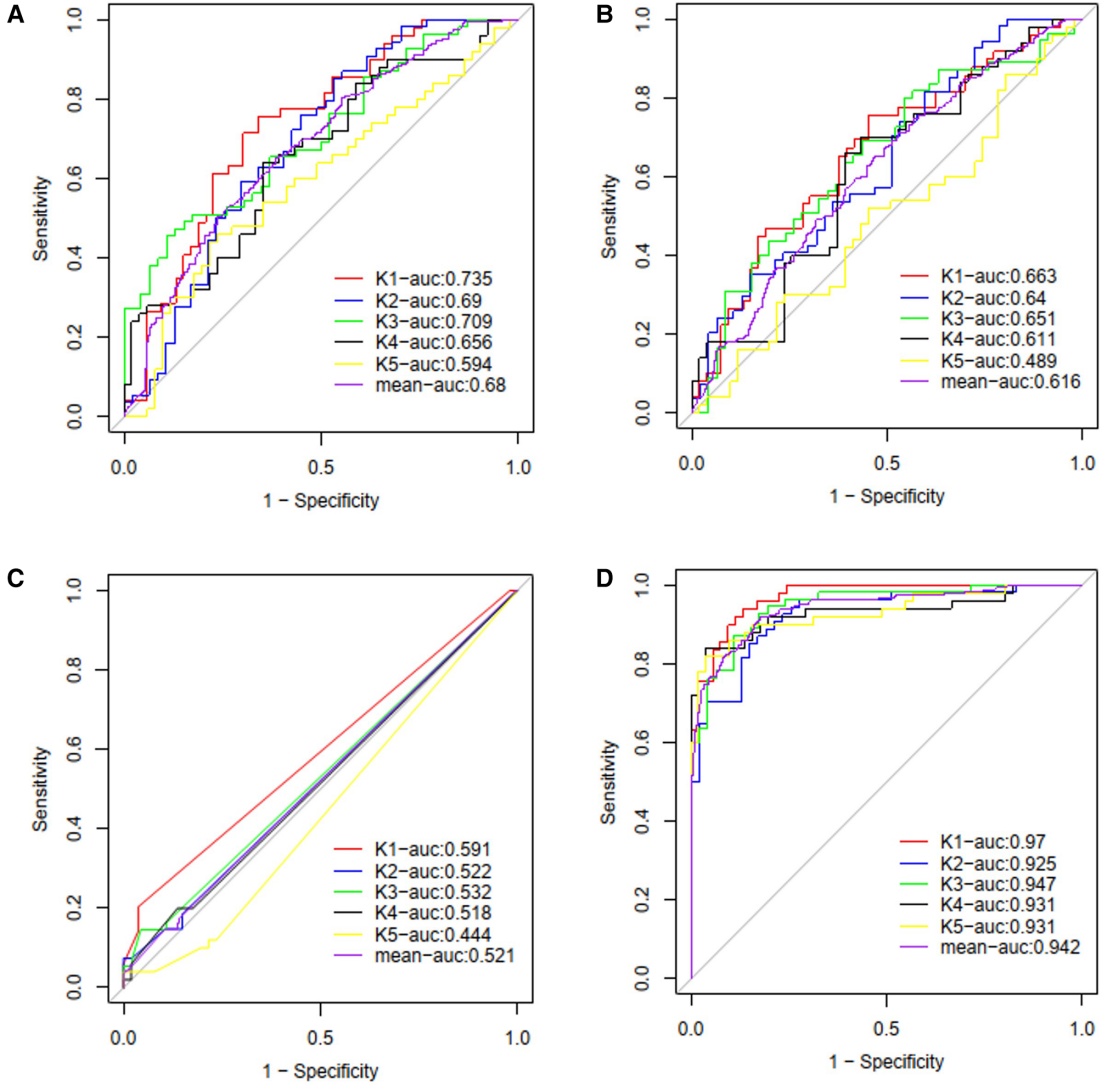
The ROC curves and average AUC of the machine learning models. K-fold cross validation (*k* = 5) was used to estimate and compare the performance of different machine learning models. After five rounds of training/validation rotation, the average AUC was calculated. (**A**) The support vector machine (SVM) model. (**B**) The logistic regression (LR) model. (**C**) The multi-layer perceptron (MLP) model. (**D**) The random forest (RF) model. ROC curve, receiver operating characteristic curve; AUC, area under the curve.

**Figure 6:**
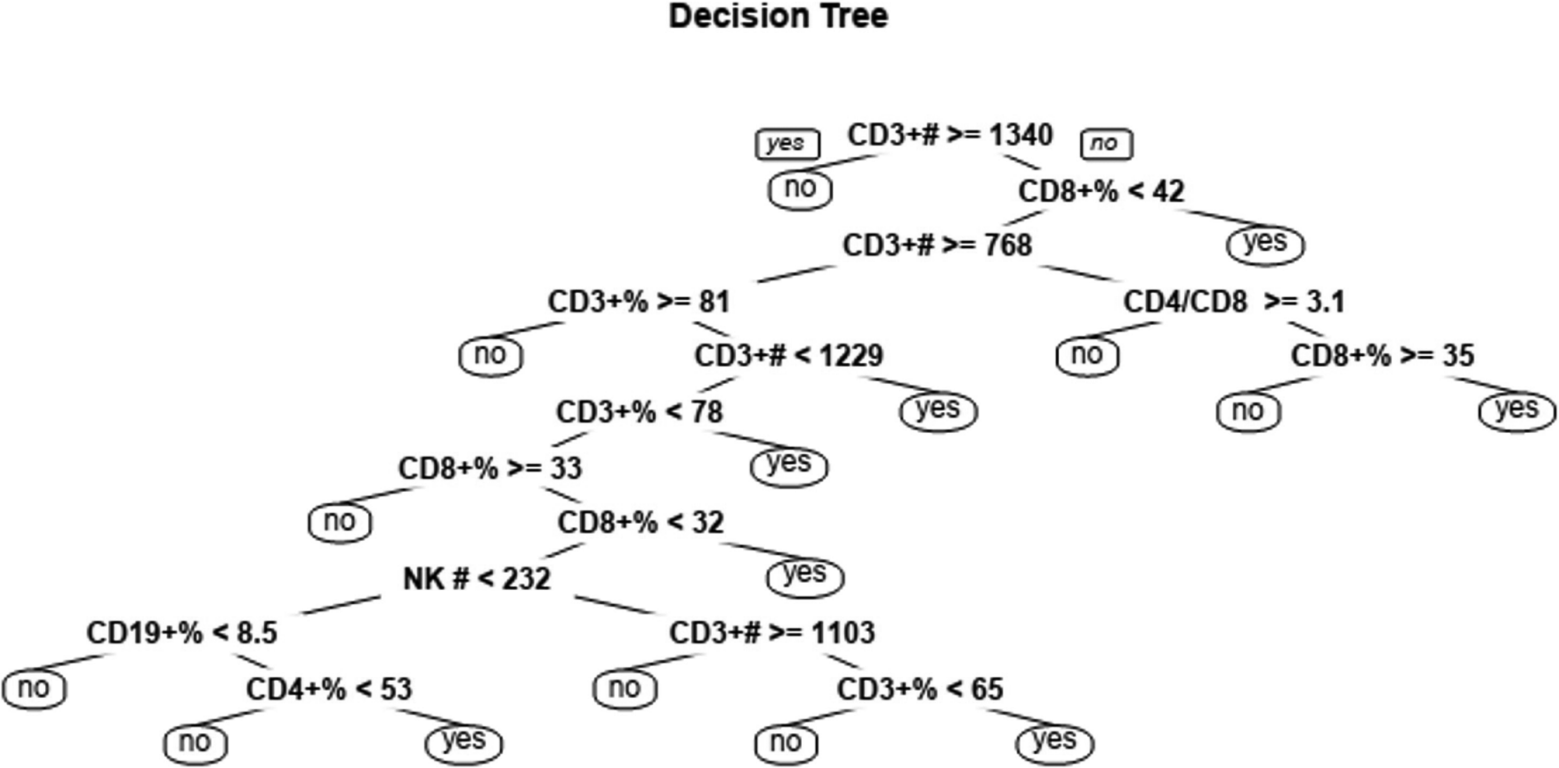
A one-tree example of random forest (RF) model. A total of ten trees were developed and one of them was shown in the figure. The final result was obtained through majority voting from ten trees.

Four ML models, namely SVM, LR, MLP, and RF, were trained and compared to enhance the predictive performance. The receiver operating characteristic curves' area under the curve (AUROC) was evaluated for each model, resulting in AUROCs of 0.68, 0.62, 0.52, and 0.94 for SVM, LR, MLP, and RF, respectively (as shown in Figure 5). The DeLong test showed a significant difference in the AUC value of the RF model compared with other ML models (*P* < 0.001, all). The RF model demonstrated the best performance among the ML models. The final RF model's result was derived from a majority vote by ten trees, and one tree of the final algorithm was displayed in Figure 6.

## Discussion

This study successfully investigated the relationship between immune status and LVH in ESKD patients. Poor immune function was observed in ESKD patients with LVH, as evidenced by decreased CD3^+^ T cell, CD8^+^ T cell, CD4^+^ T cell, and CD19^+^ B cell counts. Moreover, the study found that the number of CD3+ T cells was an independent risk factor for LVH in ESKD patients, while the number of NK cells was an independent risk factor for the severity of LVH. Using the results of the immune parameters, the RF model was able to accurately identify ESKD patients at risk of LVH. These findings might pave the way for promising therapeutic options for adverse cardiac hypertrophy and remodeling.

ESKD patients have a high risk of developing LVH, with a reported prevalence of 46.4%–65% (6, 22), which is consistent with the findings of our study (51.0%). The prevalence and severity of LVH tend to increase with the deterioration of chronic kidney disease (CKD) stages (4). During ESKD, oxidative stress and inflammation are constantly present and are considered to be closely involved in the pathogenesis of LVH. They can activate and recruit circulating lymphocytes and inflammatory myeloid cells to sites of inflammation by overproducing cytokines and increasing pro-inflammatory and oxidative stress mediators (23). This may explain the significant decrease in circulating immune cells observed in ESKD patients with LVH in our study. Furthermore, reduced renal function is closely related to dysfunctional immune system, which has a substantial impact on the development and severity of LVH in ESKD patients (24).

Circulating and heart-inﬁltrating T cell subsets played a pivotal role in the pathogenesis of adverse cardiac remodeling. T lymphocytes could be divided into CD8^+^ T cells and CD4^+^ T cells according to their function and molecular phenotype. In our study on ESKD patients, we found that the numbers of circulating T cell subsets, including CD3^+^ T cells, CD8^+^ T cells, and CD4^+^ T cells, were negatively correlated with the occurrence and severity of LVH. The decrease of circulating T cell subsets was considered to be closely related to adverse cardiac remodeling. Previous studies have shown that a decrease in circulating CD4^+^ or CD8^+^ T cells is closely associated with LVH in children with CKD and HIV patients (13, 25). Additionally, hypertensive patients with LVH have been shown to have an increased percentage of Th17 cells (9). In a study of mouse models with heart failure, CD4^+^ Foxp3^+^ Tregs were found to expand robustly in the heart and circulation for adverse cardiac remodeling, resulting in the suppression of circulating CD4^+^ T cells and systemic inflammation (14). Conversely, increased cardiac T cell infiltration has been reported in hypertrophic cardiac tissues. Numerous reports have indicated that heart-infiltrating Treg cells are negative modulators that alleviate pressure overload-induced cardiac hypertrophy and remodeling, while infiltrating Th1 and Th17 cells mainly promote the pathological development (10). NKT cells are a unique subset of T lymphocytes with properties of both T and NK cells, displaying a broad range of immune effector functions. In mice, the majority of NKT cells are invariant natural killer T (iNKT) cells, capable of recognizing lipids presented on CD1d molecules. iNKT cells modulate tissue inflammation through the secretion of diverse cytokines, growth factors, and chemokines (26, 27). iNKT cells have been shown to exhibit cardioprotective effects in murine models of myocardial infarction and hypertensive cardiac disease by reducing left ventricular remodeling through the production of anti-inflammatory cytokines like IL-4 and IL-10 (26, 28). Thus, activating NKT cells may offer a promising therapeutic strategy for hypertensive cardiac disease.

B cell subsets might play a role in the pathogenesis of cardiac remodeling in ESKD patients. Specifically, our study showed that ESKD patients with lower B cell counts were at higher risk of LVH and LVH patients with lower B cells counts have higher risk of developing severe LVH. Furthermore, in clinical settings, peripheral B lymphocyte levels were found to be negatively correlated with left ventricular mass (LVM), and patients with higher levels of B cells exhibited better heart function and a lower risk of all-cause mortality (16). Interestingly, in a mouse model of heart failure, the absence of B cells was found to result in less hypertrophy and better preservation of left ventricular function (29). In addition, a study found that the depletion of B cells by rituximab could inhibit pressure overload-induced cardiac remodeling and dysfunction in mice (30). Despite these findings, the role of B cell subsets in regulating cardiac remodeling remains controversial, and further research is needed to explore the association between B cell subsets and cardiac remodeling.

NK cell subsets might contribute to the improvement of cardiac remodeling in ESKD patients. As key players in the innate immune system and immune regulation, NK cells have a significant impact on disease severity. Specifically, our results showed that ESKD patients who developed left ventricular hypertrophy (LVH) with lower NK cell counts were at a higher risk of severe LVH. Peripheral NK cell numbers could decrease due to cytotoxic activity and infiltration in affected tissues, which could exacerbate or improve disease severity. Studies showed that a reduction in circulating NK cells could contribute to the worsening of cardiac remodeling after myocardial infarction (17). Furthermore, research had demonstrated that the crosstalk between natural killer cells and allogeneic human cardiac-derived stem/progenitor cells were beneficial for reducing inflammation and ameliorating adverse cardiac remodeling (31, 32).

Our study investigated the association between immune cell subsets and incident LVH in a cohort of patients. We found that immune dysfunction, as measured by peripheral CD4^+^ T cells, CD8^+^ T cells, CD19^+^ B cells, and NK cells, was associated with a higher risk of LVH. However, our findings differ slightly from prior studies investigating the association between immune cell subsets and cardiac hypertrophy and remodeling. Several possible explanations may account for these differences. Firstly, the composition and role of immune cell subsets may vary significantly during different stages of myocardial hypertrophy and remodeling. Most clinical and animal studies have focused on altered immune function during the acute phase of the disease, unlike the chronic phase investigated in our study. Secondly, circulating immune cells may not accurately reflect the immune status of cardiac tissue, which differs from tissue-infiltrating immune cells. Finally, the decline of specific immune cells in the circulation may be associated with their more effective migration into heart tissue due to cell differentiation and migration. The exact mechanism underlying these differences remains unclear and requires further investigation.

Our study demonstrates the utility of machine learning algorithms in identifying the association between immune cells and LVH in ESKD patients. Furthermore, these algorithms enable personalized medicine by predicting disease outcomes based on an individual's unique immune cell profile. This approach can improve the efficacy and efficiency of treatment by tailoring it to the individual patient. Overall, our study highlights the efficacy of machine learning-based approaches for analyzing complex immune system datasets and their potential to identify novel biomarkers and advance personalized medicine.

Our study had several limitations that need to be acknowledged. Firstly, this was a retrospective review of a single-center experience, and therefore, the generalizability of our findings may be limited. Future studies with large, multicenter prospective designs are needed to confirm our results. Secondly, we only investigated the numbers and proportions of peripheral immune cells, but their cytokine profiles or antigen specificities, which are crucial for assessing the function of each immune cell's role in LVH pathogenesis, were not examined. Therefore, further studies are needed to explore the functional characteristics of these immune cells. Moreover, changes in peripheral lymphocyte subsets may not accurately reflect their changes in heart tissue due to the difficulty of obtaining heart biopsy samples for LVH. Finally, our study did not assess the impact of therapeutic interventions on immune cell subsets in ESKD patients with LVH. Despite these limitations, our study provides valuable preliminary insights into peripheral lymphocyte subsets in ESKD patients with LVH and can help guide further research in this field.

## Conclusion

Our study employed machine learning models to investigate the relation between immune status and LVH in ESKD patients. Our results demonstrated that the RF model was the most effective in identifying patients at risk of LVH. With the emergence of big data, analyzing multiple parameters has become an effective approach to comprehensively understand complex diseases. Identifying the underlying pathways of this finding could offer potential targets for immunomodulatory therapy to prevent and treat LVH in ESKD patients at high risk.

## Data Availability

The raw data supporting the conclusions of this article will be made available by the authors, without undue reservation.
